# Clinical Diagnosis and Treatment of Disorders of the Bladder with Technic of Cystoscopy

**Published:** 1909-06

**Authors:** 


**Clinical Diagnosis and Treatment of Disorders of the Bladder with Technic of Cystoscopy,** by **Follen Cabot**, M.D., Professor of Genitourinary Diseases, Post-Graduate Medical School; Attending Genitourinary Surgeon, Post-Graduate and City Hospitals, New York. 8 vo, 225 pages. Forty-one illustrations, 1 colored plate. Prepaid $2.00. New York: E. B. Treat & Co., Medical Publishers, 241-243 West 23d street, New York.

Here is a small book the object of which is to teach the general practitioner methods of diagnosis and treatment of diseases of the urinary bladder. There is no floundering about in the mazes of high-flown language. It is a simple exposition of the better methods of diagnosis and treatment in conditions which, when they are met with by the general practitioner, are either ignored because of lack of understanding of their importance or entirely overlooked. The cystoscope is given careful consideration and the various instruments and the methods of their manipulation are clearly described.

The chapters on cystoscopic examination might be read to the literary advantage of other writers, for the style is clear, concise and understandable. If the general practitioner reads and takes to heart only those chapters which deal with cystitis and its causes, he will be well repaid for the time expended, for a better, clearer more comprehensive statement of the facts as bearing on this distressing condition has never been written in any book.

The illustrations deserve a word of praise. They are simply excellent and are neither exaggerated nor overdrawn. The book is small, but it is worth while.

